# Simultaneous whole‐brain and cervical spine imaging at 7 T using a neurovascular head and neck coil with 8‐channel transceiver array and 56‐channel receiver array

**DOI:** 10.1002/mrm.30450

**Published:** 2025-01-29

**Authors:** Divya Baskaran, Belinda Ding, Son Chu, Paul McElhinney, Sarah Allwood‐Spiers, Sydney N. Williams, Keith Muir, Natasha Eileen Fullerton, David Andrew Porter, Shajan Gunamony

**Affiliations:** ^1^ Imaging Centre of Excellence University of Glasgow Glasgow UK; ^2^ Siemens Healthcare Ltd Camberley UK; ^3^ MR CoilTech Limited Glasgow UK; ^4^ MRI Physics NHS Greater Glasgow and Clyde Glasgow UK; ^5^ Department of Neuroradiology, Institute of Neuroscience NHS Greater Glasgow and Clyde Glasgow UK

**Keywords:** 7 T, head and neck, neurovascular imaging, radiofrequency coils, transceiver array, ultrahigh field

## Abstract

**Purpose:**

To develop a 7T neurovascular head and neck (NVHN) coil with an extended longitudinal coverage of the brain and cervical spine, with eight transceiver (TxRx) channels and 56 receive (Rx) channels for dynamic parallel‐transmit (pTx) applications.

**Methods:**

A dual‐row transceiver array with six elements in the upper row and two elements in the lower row was designed using combined electromagnetic and circuit optimization and constructed. A 56Rx array covering the brain and cervical spine was designed and combined with the transceiver array. The performance of the 8TxRx56Rx NVHN coil such as $B_{1}^{+}$, signal‐to‐noise ratio, and g‐factor were validated in phantom and in vivo studies and compared with an in‐house 8Tx64Rx head coil. High‐resolution in vivo images were acquired with the NVHN and head coil.

**Results:**

The average $B_{1}^{+}$ in phantom while exciting the upper six channels and all eight channels are 43.45 nT/V and 45.80 nT/V, respectively, demonstrating that the available $B_{1}^{+}$ field is seamlessly distributed in the brain and/or cervical spine, depending on the chosen excitation. The 8TxRx56Rx NVHN coil increases the SNR in the cervical spine and central brain by a factor of 2.18 and 1.16, respectively, compared with the 8Tx64Rx head coil. Furthermore, it demonstrates similar 1/g‐factor performance for acceleration factors up to 5 × 5 compared with the head coil and provides diagnostic‐quality images of the brain and spinal cord in a single acquisition.

**Conclusion:**

The extended longitudinal coverage of the NVHN coil promises to improve the clinical application of the current generation of pTx 7T MRI systems with 8Tx channels.

## 1 INTRODUCTION

Current clinical neuroimaging at 1.5 T and 3 T frequently involves imaging both the brain and the cervical cord, or both, intracranial vessels at the level of the Circle of Willis and neck vessels (i.e., carotid bifurcations, internal carotid arteries, and the distal V3 and V4 segments of vertebral arteries).^1^ Clinical neuroimaging therefore frequently uses a head and neck (HN) coil to facilitate easy and fast scanning of the neuroaxis.^2^ Numerous pathologies affect the brain and cervical cord or involve the posterior fossa, brainstem and cerebellum, an area frequently not well seen at 7 T with standard commercial head coils, due to the linear decrease of radiofrequency (RF) wavelength with increasing field strength.^3^, ^4^ These include inflammatory and demyelinating conditions, such as multiple sclerosis and transverse myelitis; neurodegenerative processes involving the posterior fossa (e.g., amyotrophic lateral sclerosis or motor neuron disease) and Parkinson plus syndromes, including progressive supranuclear palsy and multisystem atrophy; vascular diseases, such as stroke and hypertensive encephalopathy; leptomeningeal and other granulomatous processes; malignant processes, including glial brain tumors, meningiomas, schwannomas, neurofibromatosis, lymphoma, paraneoplastic cerebellar degeneration; and metabolic processes such as combined subacute degeneration.^5^, ^6^, ^7^, ^8^

Imaging for pathologies such as multiple sclerosis constitutes a high workload for radiology departments. Fast‐track stroke imaging is an important requirement for any modern imaging department. A HN coil enabling imaging of the brain parenchyma to detect ischemic insult, at the same time as imaging of carotid and vertebral arteries and the Circle of Willis, with the capability of perfusion imaging, facilitates modern stroke imaging and effective treatment decision making, particularly important for planning of thrombolysis and mechanical thrombectomy. The 7T MRI with its improved signal‐to‐noise ratio (SNR), high resolution, and susceptibility artifact offers the opportunity to visualize pathologies in greater detail and earlier than at lower field strength.^9^ However, to exploit the advantages of 7T brain imaging to advance clinical use, improved visualization of the posterior fossa, simultaneous imaging of the brain and spinal cord, and simultaneous imaging of neck vessels and intracranial circulation would be highly advantageous.

Separate brain and cervical spinal cord coils are currently used to image the different anatomical regions at 7 T, because the conventional single‐row 8‐channel transmit arrays offer limited coverage, resulting in a significant drop in $B_{1}^{+}$ intensity and increased magnetic field inhomogeneity in the upper cervical spine.^10^, ^11^, ^12^ This makes clinical application in those regions challenging. Furthermore, techniques such as arterial spin labeling depend on the $B_{1}^{+}$ intensity in the upper neck to measure blood flow.^13^, ^14^ Cervical spine coils are designed to increase transmit efficiency and SNR in the neck region but lack coverage in the brain.^15^, ^16^, ^17^, ^18^ A 7T coil that covers both the brain and cervical spine is essential for various neurovascular imaging techniques such as intracranial vessel wall imaging.

Several solutions have been proposed to improve the longitudinal coverage at 7 T. A traveling wave setup for large‐volume imaging has been presented, as it provides extended coverage of the head and upper spine.^19^, ^20^ However, this setup is not widely used due to inherently weak magnetic‐field distribution. Another approach to improve the image quality in the posterior fossa combines an 8‐channel transceiver (TxRx) array with a seven‐channel receive (Rx) array,^21^ but it does not sufficiently excite the cervical spine. Other methods include the use of high‐permittivity dielectric pads^21^ and custom RF shims to enhance the $B_{1}^{+}$ efficiency in the brainstem and cerebellum.^22^ These methods increase scanning complexity and do not offer adequate cover of the upper cervical spine and neck. Dipole‐based arrays are inherently suitable for imaging larger volumes,^23^, ^24^ but the tuning stability of dipole elements is highly subject‐dependent,^25^ and the reported specific absorption rate (SAR_10g_) value is substantially higher than that of loop arrays.^26^, ^27^, ^28^ Recently, a 16TxRx array^29^, ^30^ and 16Tx64Rx^31^ have been presented for combined HN imaging at 7 T. Both arrays provide excellent coverage of the brain and cervical spine. These transmit arrays can be excited either by a 16‐channel parallel‐transmit (pTx) system or a static RF shim solution is achieved by a combination of power splitters and phase shifters if they are excited by systems with 1/2/8 transmit channels.

In dynamic full waveform pTx, the RF pulse varies with both space and time, so the pulse waveform is unique on each transmit channel.^28^ Hence a 1:1 mapping between the transmit channels of the coil and the scanner is essential to offer meticulous time‐varying control over amplitude and phase of the pTx pulse. This study aims to design an 8‐channel transmit array made of conventional loops to simultaneously excite the brain and cervical spine in 8‐channel pTx systems for dynamic pTx applications.

Previous theoretical^32^ and experimental^33^ work has predicted the need to combine loops and dipoles to approach the ultimate intrinsic SNR (UISNR) at ultrahigh field (UHF, ≥7 T). Recent studies have demonstrated that the SNR in the center of the brain can indeed be improved by combining the receive array with complementary transceiver elements such as dipoles and loopoles.^34^, ^35^, ^36^, ^37^ Avdievich et al.^26^ showed central SNR improvement of up to 20% using a 32‐element dipole/loop combined array at 7 T. At 10.5 T, Lagore et al.^38^ reported up to a 50% improvement in the central SNR by combining 120 receiver loops with 8 self‐decoupled loopole transceivers.^25^

UISNR calculations using coil elements in helmet‐shaped arrangement demonstrate a drop in SNR especially in the anterior part due to the lack of coil elements below the level of the forehead.^39^ However, at 7 T, the local transmit array surrounding the receive array extends until the level of the chin. Hence, receiving with the transmit elements could compensate for the drop in performance due to the lack of receive elements in the anterior half in helmet‐shaped arrangements. We recently demonstrated central SNR improvement while combining 56/64/96 channel receive arrays with 8/16‐element transceiver arrays made of conventional loops.^40^, ^41^ Considering this background, the transmit array was configured as an 8‐channel transceiver for improving the central SNR.

The proposed neurovascular head and neck (NVHN) coil combines the 8‐channel transceiver array with a 56‐channel receive array^42^ and aims to provide extended longitudinal coverage of the brain, posterior fossa, upper cervical spine, internal carotid arteries, carotid bifurcations, and distal segments of vertebral arteries. The 8TxRx array was optimized using electromagnetic and circuit co‐simulation methods. The numerical model of the coil was used to analyze the $B_{1}^{+}$ distribution and SAR performance in realistic human body models. After constructing the optimized coil, $B_{1}^{+}$ field distribution, SNR, and 1/g‐factor were measured in both phantom and in vivo and compared with the in‐house‐built 8Tx64Rx head coil.^10^, ^43^ Finally, we acquired high‐resolution three‐dimensional (3D) in vivo images of healthy volunteers to confirm the ability to produce images of diagnostic quality, covering the brain, upper cervical spine, and neck vessels, in a single acquisition.

## 2 METHODS

### 2.1 Numerical simulation

**FIGURE 1:**
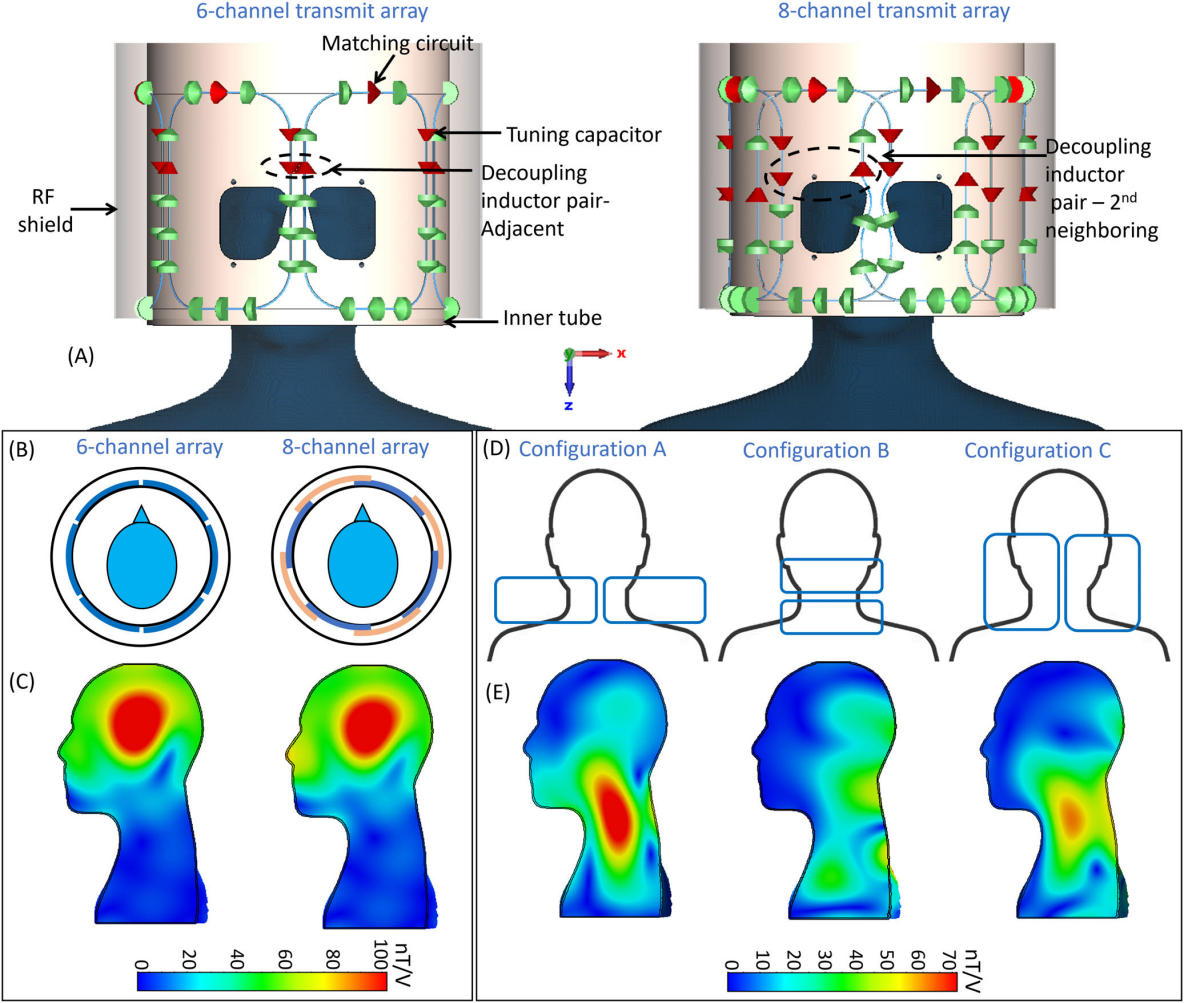
(A) Front view of the numerical model shows the 6‐channel and 8‐channel transmit arrays. Green and red markers indicate the lumped element and discrete port of the loop, respectively. (B,C) Layout of the 6‐channel and 8‐channel arrays and their respective field distribution in the central sagittal slice of the head and shoulder (HS) phantom in circularly polarized (CP) mode. In the 8‐channel configuration, the adjacent overlapping loops are shown in blue and orange, to distinguish them from each other. For better visualization, they are displayed in two different layers. (D) Illustration of three analyzed configurations of the lower two elements. (E) Its Respective $B_{1}^{+}$ field distribution in the HS phantom obtained at the best phase difference between them.

Two 8‐channel transmit arrays were simulated: a reference array consisting of eight elements in one row and the proposed design consisting of six elements in the upper row and two elements in the lower row. The upper and lower rows were optimized separately and then combined (Figure 1). The 3D electromagnetic and circuit co‐simulation was carried out using the time domain solver *CST Studio* suite 2021 (Dassault Systems, Paris, France), which uses the finite‐integration technique.^44^ The transmit arrays were made of two concentric fiberglass tubes $\left(\varepsilon_{r}=4.3,\tan\delta =0.025\right)$ consisting of an inner tube (290‐mm inner diameter, 2‐mm wall thickness) and an outer tube (354‐mm inner diameter, 2‐mm wall thickness). The transmit elements were mounted on the outer surface of the inner tube, and a local RF shield made of continuous copper ($\sigma =5.8\times 10^{7}S/m,\rho =8.93\times 10^{3}\;\mathrm{kg}/m^{3}$) was attached to the inner surface of the outer tube to minimize radiation loss (Figure 1A). The numerical model also included the scanner RF shield, realized as a 1‐mm‐thick copper tube with an inner diameter of 640 mm and a length of 1580 mm.

The reference 8‐channel array consisted of eight overlapped loops^10^ spaced at an angular separation of 45°, whereas the proposed 6‐channel array consisted of six non‐overlapped loops separated by 60° (Figure 1A,B). The dimensions of the loop in both arrays were similar, with a width of 140 mm and a length of 210 mm. The adjacent elements in the 8‐channel array were critically overlapped,^45^ and the next‐neighboring elements were decoupled with a pair of decoupling inductors modeled as two coupled inductors of equal value with mutual inductance and a coupling factor of −0.5.^46^ All the adjacent elements in the 6‐channel array were decoupled with a decoupling inductor pair. There were 16 capacitors (15 fixed and 1 variable) evenly distributed in each loop of the 6‐channel array, whereas each 8‐channel array element consisted of 14 evenly distributed capacitors (13 fixed and 1 variable), and the capacitors were connected using 2‐mm copper wire (Figure 1A). The fixed capacitors were modeled as lumped elements, and the matching circuit, variable capacitors, and decoupling inductors were modeled as ports. The variable capacitor in the matching and tuning circuit had a range from 1 to 9 pF. Each lumped element included series resistance and inductance values from the component datasheet. Two cutouts of 70 × 65 mm^2^ were added in front of the eyes.

Simulations were performed on a workstation with an Intel Xeon gold 6244 CPU processor (GPU acceleration using Nvidia, 128 GB RAM, and 16 cores; Dell Technologies Inc., Round Rock, TX, USA). The frequency was swept between 250 MHz and 350 MHz with a convergence criterion of −40 dB. The typical mesh consisted of 100 million mesh cells. Both the 8‐channel and 6‐channel arrays were tuned to 297.2 MHz and matched to 50 ohms while loaded with a head and shoulder (HS) phantom filled with tissue equivalent solution ($\varepsilon_{r}=52.1$ , σ = 0.41 S/m).^46^, ^47^ Initial simulation results in circularly polarized (CP) mode (Figure 1C) showed that the efficiency of the 6‐channel and 8‐channel coil was comparable, allowing the remaining two channels to be moved to the posterior half of the neck to extend the longitudinal coverage. We also analyzed the performance of the lower two elements in three different configurations, A, B, and C (Figure 1D). Each element was excited with 0°, −30°, −60°, and −90° phase difference. Configuration A resulted in a higher $B_{1}^{+}$ field intensity in the neck region compared with the other configurations (Figure 1E). The two loops in this configuration were 210‐mm wide and 120‐mm in length (along Z) and wrapped 180° around the back of the neck.

**FIGURE 2:**
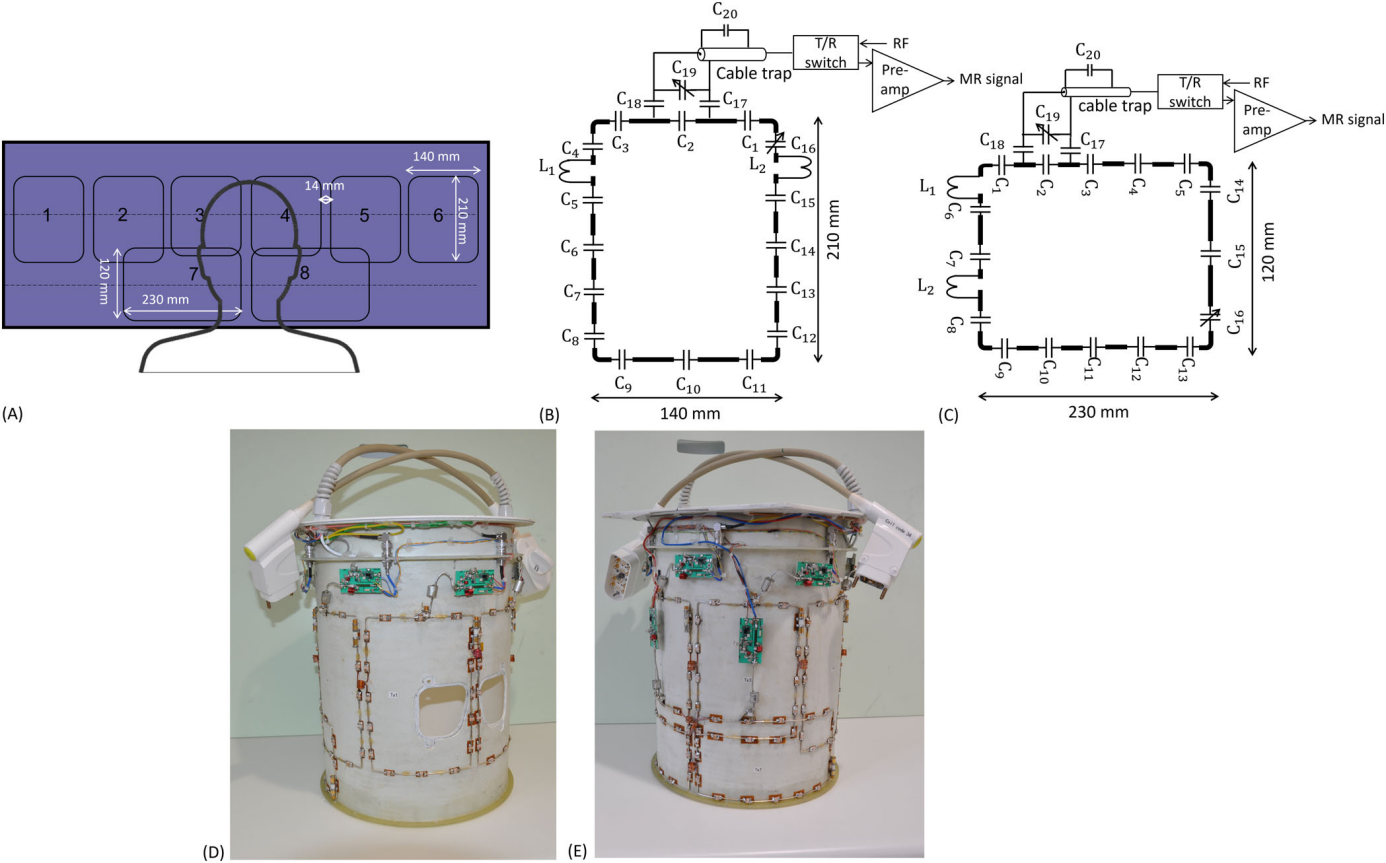
(A) Illustration of the 8TxRx array arranged in two rows. The upper row consists of six transceiver elements, and the lower row consists of two transceiver elements. The length of TxRx3 and TxRx4 was 185 mm, and all other loops in the top row were 210 mm. Schematics of the transceiver elements in upper row (B) and lower row (C). Each element is connected to transmit/receive (T/R) switches via a shielded cable trap. Constructed 8TxRx array with T/R switches: front view (D) and back view (E).

Thus, the proposed 8TxRx array consists of two rows with six elements in the upper row (TxRx1–6) and the remaining two elements (TxRx7–8) placed in the lower row (Figure 2A). The critical overlap distance between the two rows was optimized in electromagnetic and circuit simulation by adjusting the length of TxRx3 and TxRx4. The diagonal elements TxRx4, TxRx7 and TxRx3, TxRx8 were decoupled with decoupling inductor pair. The optimized coil was tuned to 297.2 MHz and matched to 50 ohms while loaded with the HS phantom. The relative phase of Channels 7 and 8 was optimized in *MATLAB* (R2023b; MathWorks, Natick, MA, USA) to maximize the $B_{1}^{+}$ field distribution in the neck region without comprising the field distribution in the head region. The optimized pseudo‐CP phases of the 8TxRx array are [−30°, −90°, −150°, −210°, −270°, −330°, −72°, −216°]. The S‐parameters and $B_{1}^{+}$ field distribution, while exciting only the upper 6 elements and all 8 elements in pseudo‐CP mode, were calculated.

The coil was then loaded with Duke, Ella, and Gustav voxel models^48^ with an isotropic resolution of 1 mm from the virtual family truncated until the chest for SAR assessment. For each voxel model, simulations were carried out at three different positions (isocenter [*z* = 10 mm and *z* = 20 mm]), and Q‐matrices were generated. These matrices were concatenated and compressed to generate a virtual observation point file and loaded in the scanner for SAR management.^49^

### 2.2 Construction of the proposed 8‐channel transceiver array

The transceiver and receive array were constructed on two separate coil housing assemblies. The transceiver array housing had two concentric rapid‐prototyped fiberglass tubes with the same dimensions as in the numerical model. The local RF shield was realized using a slotted two‐layered flexible polyamide PCB (Multi‐CB, Munich, Germany) with an 18‐μm‐thick copper layer and was attached to the inner surface of the outer tube using double‐sided tape (3M 9086; RSpro, Corby, UK). The transceiver elements were implemented on the outer surface of the inner tube as per the equivalent circuit (Figure 2B,C). Pads to solder the capacitors were glued with double‐sided tape on the fiberglass tube in the same locations as in the numerical model. Each resonant loop consisted of 15 fixed capacitors ($C_{1}$ to $C_{15}$ [6.2 pF], DLC70C; Dalicap, Dalian, China) and a variable capacitor C_16_ (5610; Johanson, Boonton, NJ, USA). The matching circuit consisted of fixed capacitors C_17_, C_18_ (39 pF) and a variable capacitor, $C_{19}$ (52H02; Johanson). The solder pads are joined together by 2‐mm silver‐plated copper wire (Wires, Chelmsford, UK) to complete the loop.

The decoupling inductors ($L_{1}$ and $L_{2}$ ) were implemented as two counter‐wound inductors (1.25‐mm‐diameter enameled copper wire [357–794; RS PRO, Corby, UK]) aligned opposite to each other (Figure 2D,E). Each channel was connected to custom‐built transmit/recieve (T/R) switches^50^ (Figure 2B,C), and the transmit and receive functions were separated by appropriately biasing the pin diodes (MA4P7446; MACOM, Lowell, MA, USA). The T/R switch unit contains a pre‐amplifier (WMA7RD; Wantcom, Chanhassen, MN, USA) in the receiver path. A shielded cable trap tuned to 297.2 MHz was used to connect each loop to its respective T/R switch. The length of the cable trap and matching capacitor ($C_{2}$ and $C_{19}$ ) were adjusted to achieve pre‐amplifier decoupling in the receive condition. The constructed 8TxRx array with the T/R switches is shown in Figure 2D,E.

### 2.3 Construction of the proposed 56‐channel receive array

**FIGURE 3:**
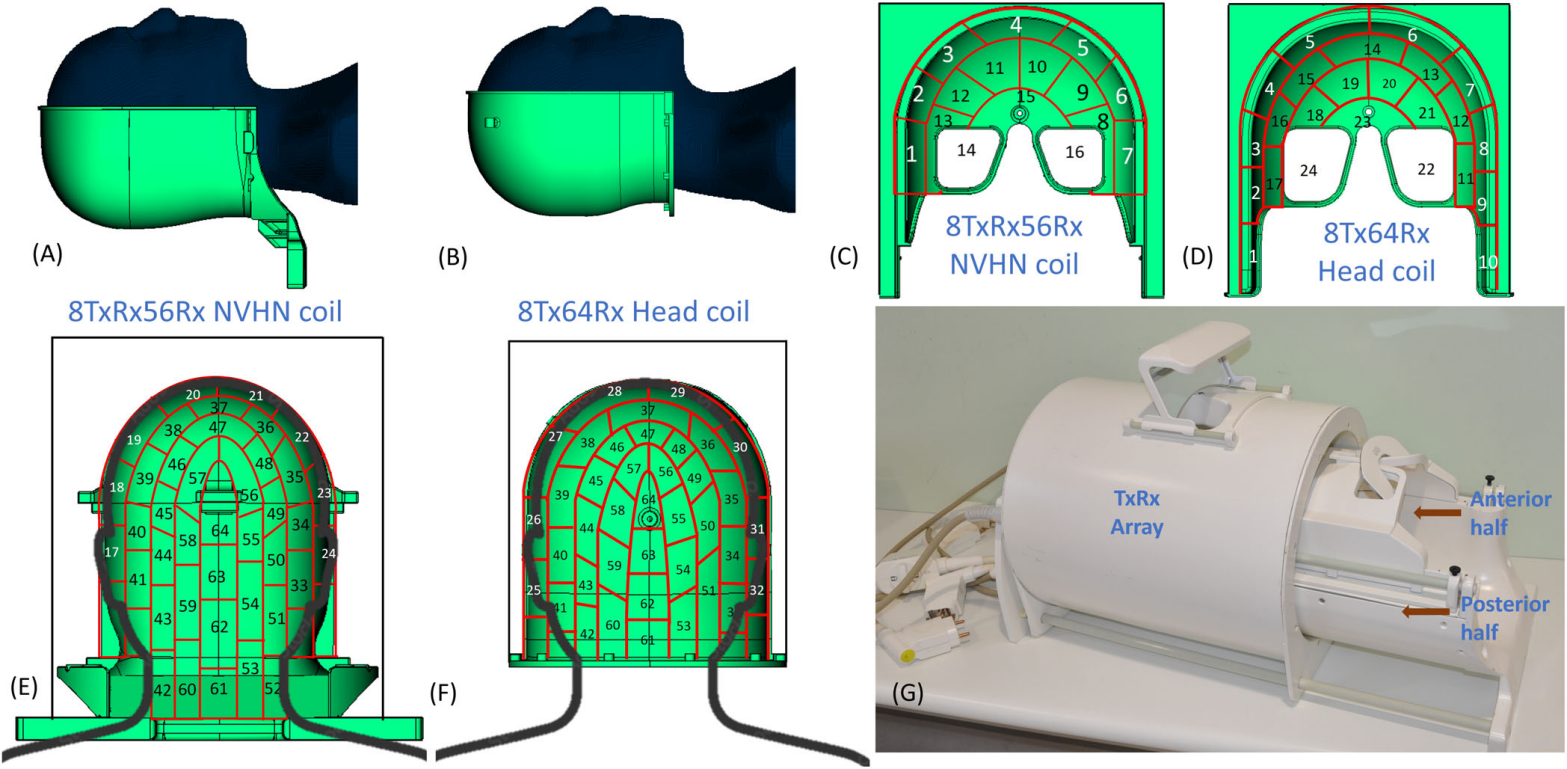
(A,B) Posterior receive housing of 8TxRx56Rx NVHN coil and 8Tx64Rx head coil with HS phantom. (C) Anterior half of NVHN coil with 16 receiver elements arranged in three rows and two eye loops (Rx14 and Rx16). (D) Anterior half of the head coil with 24 receiver elements arranged in four rows with two eye loops (Rx24 and Rx22). (E,F) Posterior half of the NVHN and head coil with 40 receive elements arranged in five rows. NVHN coil element numbers are mapped to scanner receiver channel number. Anterior elements are connected to Rx1 to Rx16. Transceiver elements are connected to Rx25 to Rx32, and posterior elements are connected to Rx17 to Rx24 and Rx33 to Rx64. (G) Constructed final coil assembly.

The receive helmet was designed to be close‐fitting and consisted of an anterior and a posterior half. Figure 3A–F demonstrates the difference between the receive arrays of the NVHN coil and the reference 8Tx64Rx head coil. The difference is primarily in the posterior half, in which the NVHN coil is extended to cover the neck (Figure 3A,E), whereas only the head region is covered in the reference head coil (Figure 3B,F). Although the internal dimensions of the two helmets are the same in left/right (186 mm) and in anterior/posterior direction (220 mm), in the head/foot direction the posterior half of the NVHN coil is longer by 74 mm compared with the reference head coil (294 vs. 220 mm).

The helmet was 3D‐printed using selective laser sintering technique (PA2210FR; Ogle models, Letchworth, UK), and the external surfaces were painted with bio‐compatible paint. In the anterior half, there were 16 elements in the NVHN coil (Figure 3C) and 24 elements in the head coil (Figure 3D). Both receive arrays have 40 elements in the posterior half (Figure 3E,F). The receive elements were arranged in a staggered pattern, with each element geometrically overlapping with the adjacent elements in the same column and with two elements in the neighboring column to reduce mutual coupling.^45^ The construction of the transmit and receive array of reference head coil is briefly explained in Feinberg et al.^41^ The 16 elements in the anterior half of NVHN are arranged in three columns plus the two large eye loops. In the posterior half, 40 elements were evenly distributed in five columns, with 10 of those elements in the neck region. Most coil elements are rectangular in shape, but some had shorter or curved segments depending on the profile of the helmet. The average dimension of the receive element is 50 × 60 mm^2^.

Each receive element consisted of an input board with a matching and active detuning circuit, two fixed capacitors (7.5 pF; Dalicap, Dalian, China), and a variable capacitor (27293NM‐007; Johanson, Boonton, NJ, USA) to tune the loop to 297.2 MHz.^51^ A 1.25‐mm fine silver wire (metalclays4you, Dalkeith, UK) was used to connect these capacitors and close the loop. For secondary safety in case of failure of the active detuning circuit, a low‐resistance fuse with a nominal current rating of 315 mA was added in series. Each loop was connected to low‐impedance pre‐amplifiers (WMA7RD; WanTcom, Chanhassen, MN, USA) by a shielded coaxial trap to suppress the common mode current. A series capacitor at the input of the preamplifier was adjusted to achieve preamplifier decoupling.

A sliding mechanism was implemented for comfortable patient positioning. The anterior half as well as the transmit assembly could be moved back and forth on sliding rods and locked in place. The completed coil (Figure 3G) also included a look‐out mirror, which is especially beneficial for claustrophobic subjects to see the operator while scanning.

### 2.4 Bench measurements

Bench measurements were performed using a two‐port ZND series network analyzer (Rohde and Schwarz, Memmingen, Germany). The tuning, matching, and decoupling of the TxRx array elements were evaluated by measuring S‐parameters while loading the coil with the HS phantom.^46^ For S‐parameter measurement of the transceiver array, the T/R switch was switched to transmit mode, and all 56 Rx channels were actively detuned. Except for the channel under measurement, all other channels were terminated with 50 ohms.

Pre‐amplifier decoupling and active detuning of the receive channels were measured using a lightly coupled double‐loop H‐field probe. The sensitivity of each receive chain was measured using a single‐loop H‐field probe. Custom‐built test rigs were used to control the switching and biasing of the PIN diodes and preamplifiers.

### 2.5 Coil validation

All MR measurements were conducted in a whole‐body 7T scanner (MAGNETOM Terra; Siemens Healthineers AG, Erlangen, Germany). The transmit performance of the NVHN coil and reference head coil was characterized by single‐channel and combined $B_{1}^{+}$ mapping using the HS phantom and was compared with simulation results. Single‐channel relative $B_{1}^{+}$ maps were obtained by acquiring low‐flip‐angle FLASH images transmitting on one channel at a time before being paired with the single combined absolute $B_{1}^{+}$ map. Combined $B_{1}^{+}$ map was acquired using a saturation‐recovery turbo‐FLASH sequence^52^ with the following parameters: repetition time (TR) = 564 ms, acquisition time (TA) = 20 s, echo time (TE) = 1.9 ms, imaging bandwidth (BW) = 490 Hz/px, in‐plane resolution = 2.2 mm, slice thickness = 5 mm; 15 sagittal slices; and matrix size = 128 × 128. The used sequences are based on an in‐house well‐established coil validation protocol. Combined $B_{1}^{+}$ maps were acquired twice in pseudo–CP mode: first with all eight channels and then with only the top six channels.

### 2.6 In vivo imaging

Coil safety was validated as per the recommendations in Hoffman et al.^53^ and was approved for human imaging by the local safety and ethics committee. Two healthy volunteers were included in this study, and all in vivo images were acquired in pseudo‐CP mode. Both volunteers were scanned with the reference 8Tx64Rx head coil and the NVHN coil.

For SNR and 1/g‐factor mapping, non‐accelerated gradient‐recalled echo images consisting of 75 transversal slices were acquired in healthy volunteer with the following parameters: TR = 6000 ms, TE = 3.82 ms, flip angle (FA) = 40°, resolution = 4 × 4 × 4 mm^3^, matrix size = 58 × 58, and BW = 990 Hz/Px. A noise scan was acquired with matching parameters but with 0 V and a reduced TR of 500 ms. To correct for variations in transmit sensitivity, $B_{1}^{+}$ maps were acquired with actual flip‐angle imaging (AFI) with matching imaging volume.^54^ The AFI method offers an increased range of linearity and was chosen as the optimum technique for this application. Other imaging parameters for AFI include TR1/2 = 10/100 ms, TE = 2.04 ms, FA = 60°, in‐plane resolution = 4 mm, slice thickness = 4.16 mm, 72 slices, matrix size = 58 × 58, imaging bandwidth = 980 Hz/Px, and GRAPPA acceleration factor = 2 × 2. Raw k‐space data from the gradient‐recalled‐echo signal, noise scans, and DICOM images from AFI were exported offline and postprocessed in *MATLAB*. SNR maps were calculated as described in Refs. 45, 55, and 56.

In vivo combined $B_{1}^{+}$ map using AFI, two‐dimensional (2D) T_2_‐weighted turbo spin echo, and T_1_‐weighted MP2RAGE sequence^57^ were acquired in a healthy volunteer. The sequence parameters of T_2_‐weighted turbo spin echo are TR = 5000 ms, TE = 68.45 ms, in‐plane resolution = 0.46 mm, slice thickness = 3 mm, 22 sagittal slices, matrix size = 640 × 630, refocusing FA = 135°, BW = 290 Hz/px, and TA = 5 min 45 s. Sequence parameters of MP2RAGE are as follows: resolution = 0.8 × 0.8 × 0.8 mm^3^, 384 slices, GRAPPA acceleration factor = 4, TR = 5000 ms, TE = 1.92 ms, BW = 495 Hz/px, FA = 4°, and TA = 6 mins 20s. The volunteer was again assessed with a high‐resolution T_1_‐weighted FLASH gradient‐echo sequence (TR/TE = 35/2 ms, BW = 475 Px/Hz, matrix size = 320 × 260, in‐plane resolution = 0.9 mm, slice thickness = 0.75 mm; 224 sagittal slices, and TA = 8 min 40s) and phase contrast MRA (PC‐MRA) in the coronal orientation with the following parameters: TR = 57.4 ms, TE = 8.38 ms, FA = 20°, in‐plane resolution = 0.5 mm, slice thickness = 0.5 mm; matrix size = 400 × 318, 144 slices, and velocity encoding = 15 cm/s.

## 3 RESULTS

### 3.1 Phantom results

**FIGURE 4:**
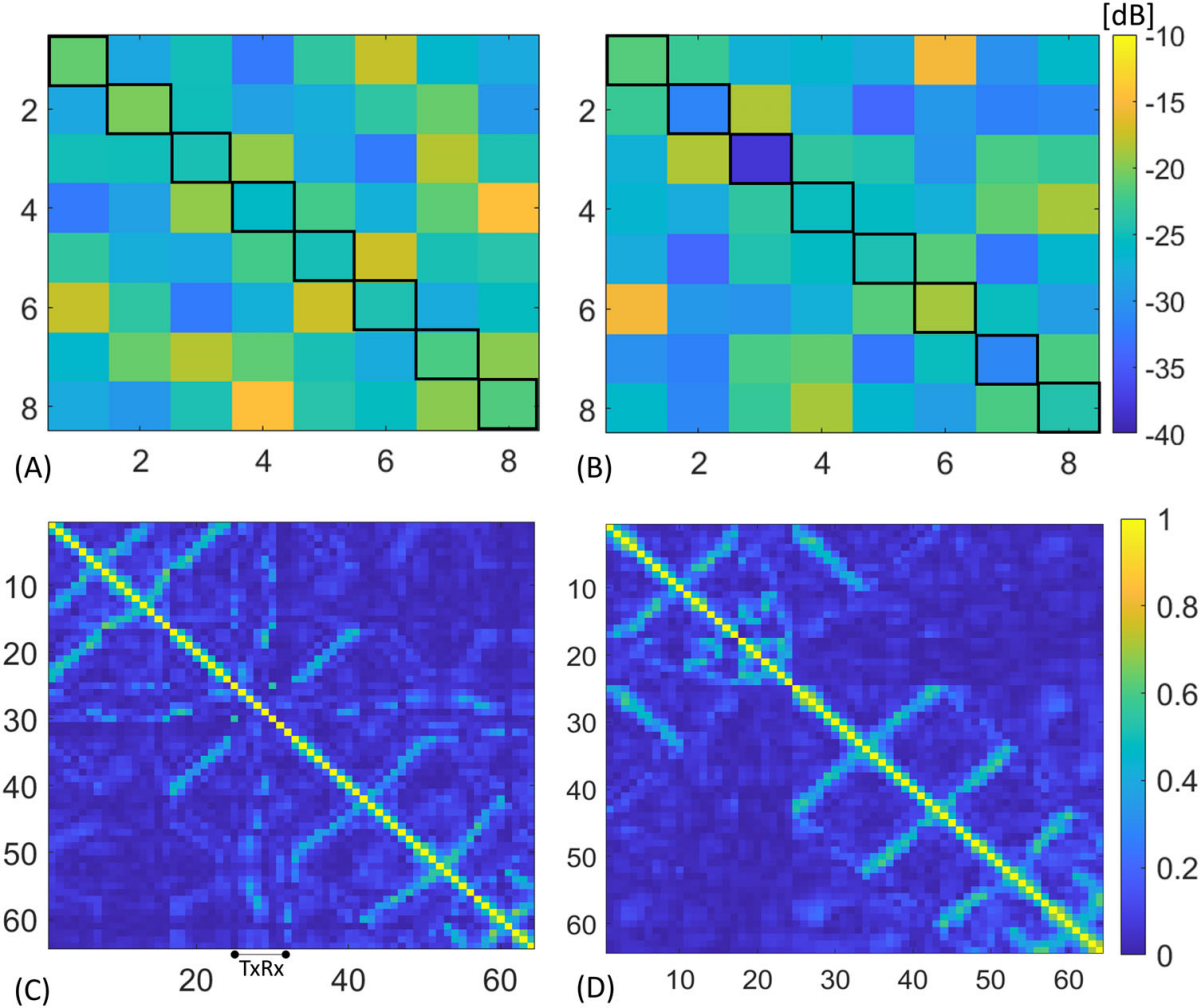
S‐parameter and noise correlation matrices for the NVHN coil while loaded with a head and shoulder phantom. (A) Simulated S‐parameter matrix for the 8TxRx array. (B) Measured S‐parameter matrix for the 8TxRx in the presence of the actively detuned 56Rx array. (C) Noise correlation matrix of 8TxRx56Rx NVHN coil. Transceiver elements correspond to Channels 25 to 32. (D) Noise correlation matrix for the 64‐receive head coil. The average off‐diagonal correlation coefficient is 12.6% and 14.8% for the NVHN and head coil, respectively.

Figure 4A–C shows the simulated, measured S‐parameter matrix of the constructed 8TxRx and the noise correlation matrix of the 64 Rx channels of the NVHN array. All transmit channels were matched to better than −19 dB in both simulation and measurement, and decoupling values ranged from −15.46 to −38.1 dB. The coupling between the upper and lower rows was less than −18.7 dB. The highest coupling was observed between TxRx1 and TxRx6, which was −17.6 and −15.46 dB in the simulation and measurement, respectively. This is primarily due to the large eye cutouts in the RF shield above Channels 1 and 6. The noise correlation of the off‐diagonal elements of the 8TxRx56Rx NVHN coil ranged from 0.04% to 67.5%, with an average of 12.6% (Figure 4C). The average noise correlation of the off‐diagonal elements of the 8Tx64Rx head coil was 14.8% (Figure 4D).

The simulated 2D average $B_{1}^{+}$ field in the sagittal midplane on the HS phantom while exciting the upper 6 channels and all 8 channels of the NVHN coil in pseudo‐CP mode was 48.76 and 46.93 nT/V, respectively. The simulated peak SAR_10g_ for 1W excitation in Duke, Ella, and Gustav models for pseudo‐CP excitation at the isocenter position of NVHN coil was 0.21, 0.28, and 0.38 W/kg, respectively. The peak SAR_10g_ among the nine simulations was 0.38 W/kg, and the k‐factor was set to 1, providing a safety factor of 2.6.

**FIGURE 5:**
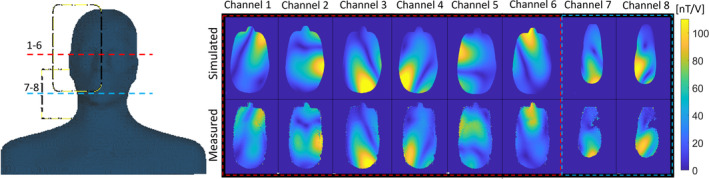
Simulated and measured single‐channel magnitude maps of each transceiver element in the head and shoulder phantom. The distribution is shown for a transverse plane in the middle of the head for Channels 1–6 and in the upper neck region for Channels 7–8. These two slice positions relative to the phantom are shown as red and blue dashed lines on the left of the figure.

**FIGURE 6:**
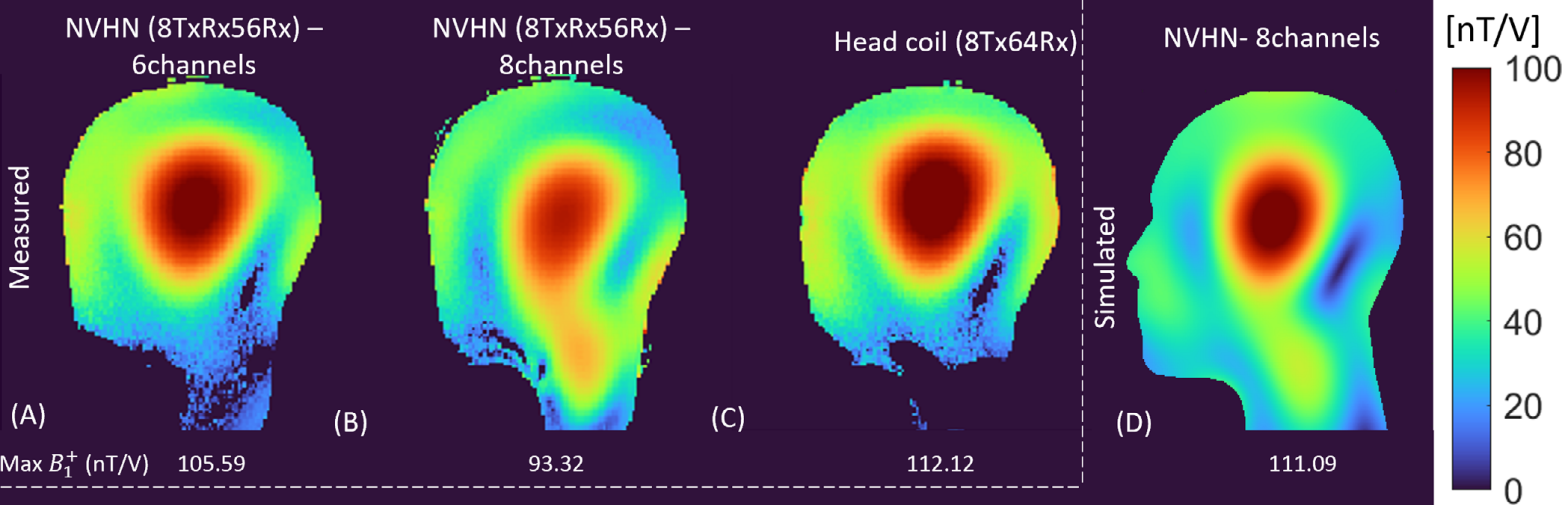
$B_{1}^{+}$distribution of the NVHN coil in the sagittal mid‐plane of the HS phantom in pseudo–CP mode. (A,B) While exciting, the upper six channels alone (A) and all eight channels of the array (B). (C) Measured combined $B_{1}^{+}$ of the 8Tx64Rx head coil in CP mode. (D) Simulated $B_{1}^{+}$ distribution while exciting all eight channels of the proposed NVHN coil.

In Figure 5, the measured single‐channel $B_{1}^{+}$ distribution of the NVHN coil is compared with the simulated data. The spatial distribution of the $B_{1}^{+}$ field in the simulated and measured plots is in good qualitative agreement with minor deviations in some channels. The average $B_{1}^{+}$ distribution calculated in the sagittal midplane of the HS phantom acquired with a slice thickness of 5 mm (Figure 6A,C) while exciting the upper 6 channels and all 8 channels in pseudo‐CP mode are 43.45 and 45.80 nT/V, respectively. All losses are calibrated until the coil input, and the total input power remains the same for all the measurements. The measured peak $B_{1}^{+}$ in the head region while exciting all 8 channels was 93.32 nT/V, which is 15.9% less than in simulation. This difference can be attributed to the underestimation of resistive losses associated with capacitors, inductors, connecting wires, and solder joints in the simulation. The peak $B_{1}^{+}$ in the head region while exciting 6 channels of NVHN coil is only 5.8% lower than the reference 8‐channel head coil (Figure 6A,C). Exciting the lower two channels (TxRx7 and TxRx8) in the NVHN coil increases the average $B_{1}^{+}$ distribution in the neck region in the central sagittal slice by a factor of 2.9 (Figure 6A,B). Characterization of the reference 8‐channel transmit array can be found in Refs. 10 and 43.

### 3.2 In vivo results

**FIGURE 7:**
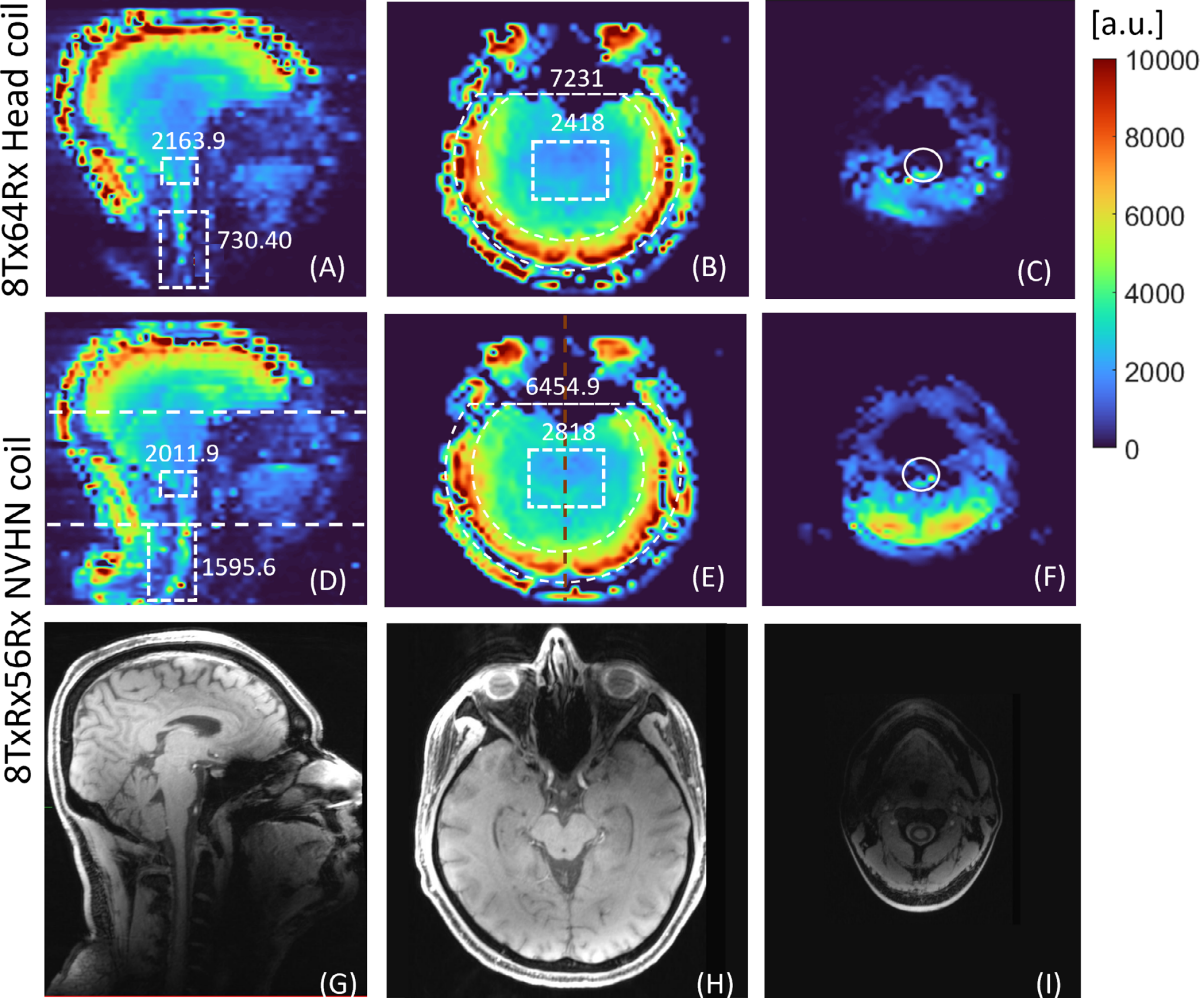
Measured SNR map in a healthy subject with the conventional head coil (*top*) and proposed NVHN coil (bottom). (A,D) SNR maps in the sagittal midplane. (B,E) SNR maps in a transverse plane through the brain. (C,F) SNR maps in a transverse plane in the neck region. All SNR maps have been normalized by the $B_{1}^{+}$ map. The locations of the transverse and sagittal planes are shown in (D) and (E) as white and brown dashed likes, respectively. The average sensitivity values within the brainstem, cervical spine, and center and periphery of the brain region calculated within white dashed rectangles within the slice in (A)–(D) are also shown. The SNR in the cervical spine, brain stem, and center and periphery of the brain averages over 220, 64, 204, and 3248 voxels. The spinal cord region is highlighted with a white solid line in (C) and (F). The respective magnitude images showing the anatomy are presented in (G)–(I).

**FIGURE 8:**
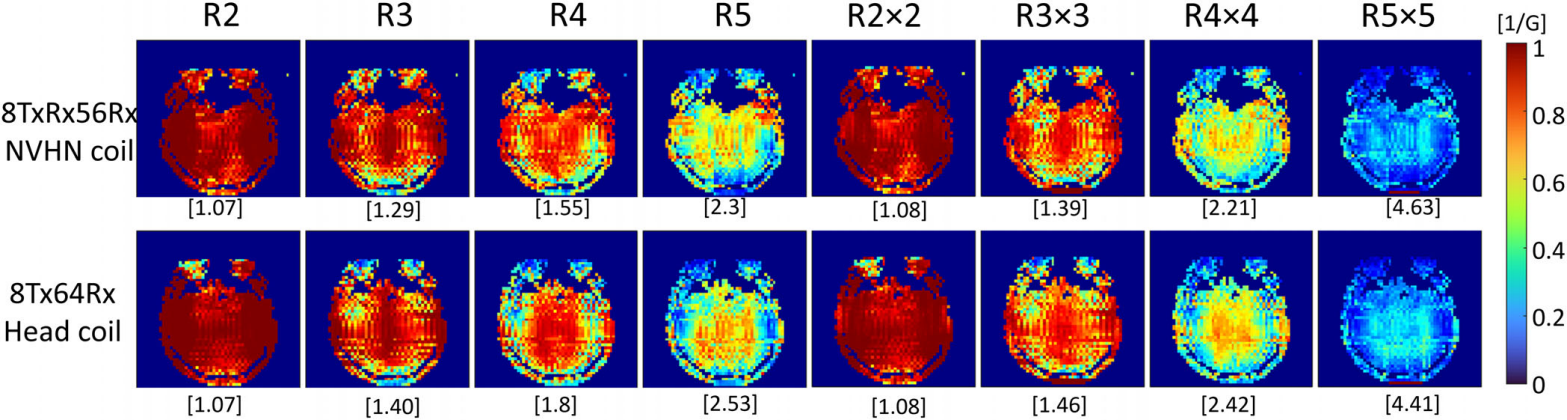
Inverse g‐factor maps in a transverse slice through the brain of a healthy subject. Comparison between NVHN coil and conventional head coil for different acceleration factors. The mean g‐factor for the slice is shown below the map in each case.

In vivo SNR maps shown in Figure 7 demonstrate the extended coverage of the cervical spine and the increase in the central SNR with the NVHN coil compared with the head coil. SNR gain in the cervical spine was by a factor of 2.16, and the gain in central SNR due to the transceiver function was by a factor of 1.16. The SNR in the periphery was 10.7% lower than that of the head coil due to the larger loop size. Furthermore, the SNR in the brainstem region was 7% lower for the NVHN coil, indicating the need for refinement of the receive element dimension and distribution in the neck region. Figure 8 demonstrates the inverse g‐factor maps of the NVHN and head coil with one‐dimensional (1D) and 2D acceleration. From the mean values of g‐factors, it is evident that both coils offer similar acceleration performance intracranially.

**FIGURE 9:**
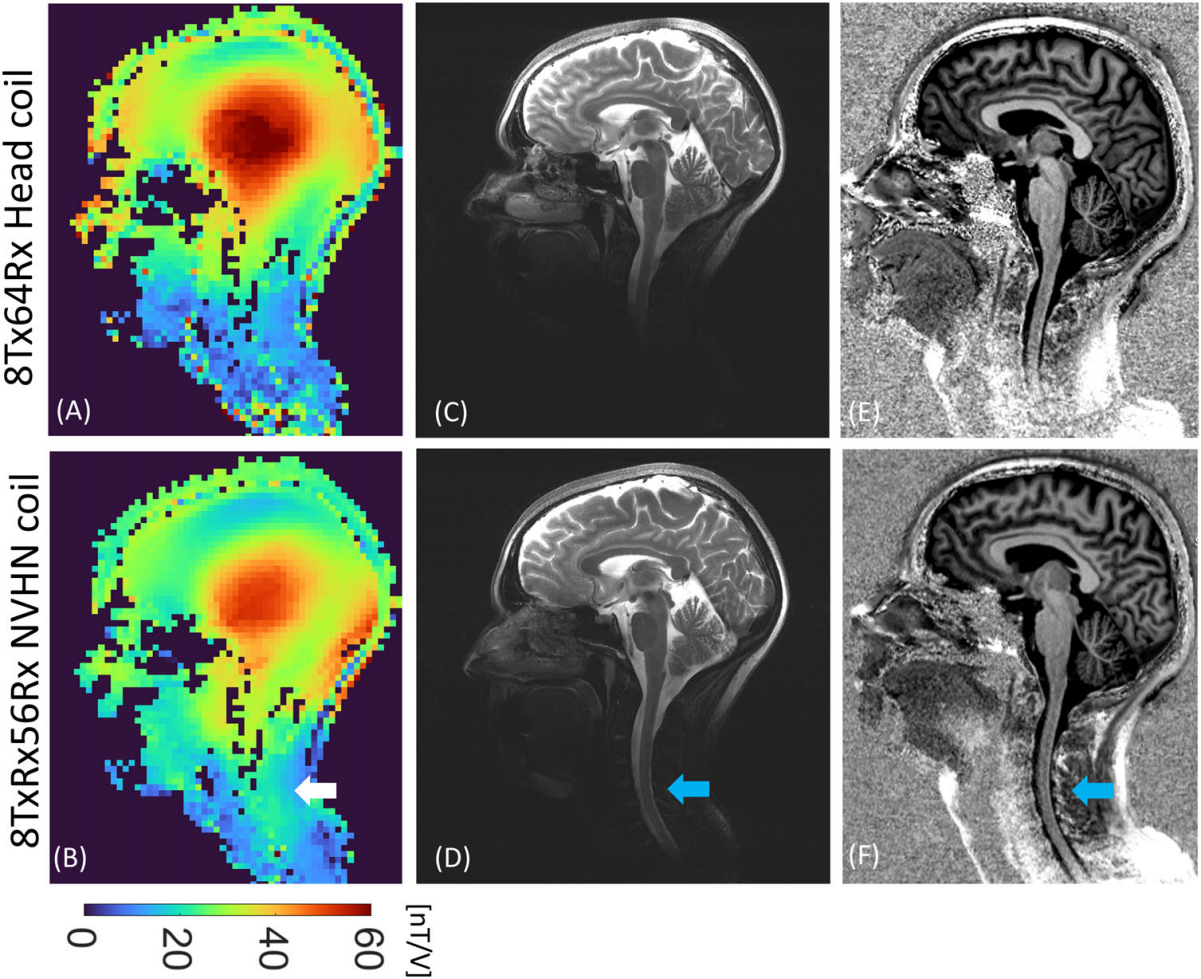
In vivo images of a healthy volunteer in the sagittal midplane acquired using the proposed NVHN coil and head coil. (A,B) Combined $B_{1}^{+}$ map. (C,D) Two‐dimensional T_2_‐weighted turbo spin echo. (E,F) MP2RAGE sequence. The arrow in (B), (D), and (F) shows the elongated longitudinal coverage offered by the NVHN coil compared with the head coil.

**FIGURE 10:**
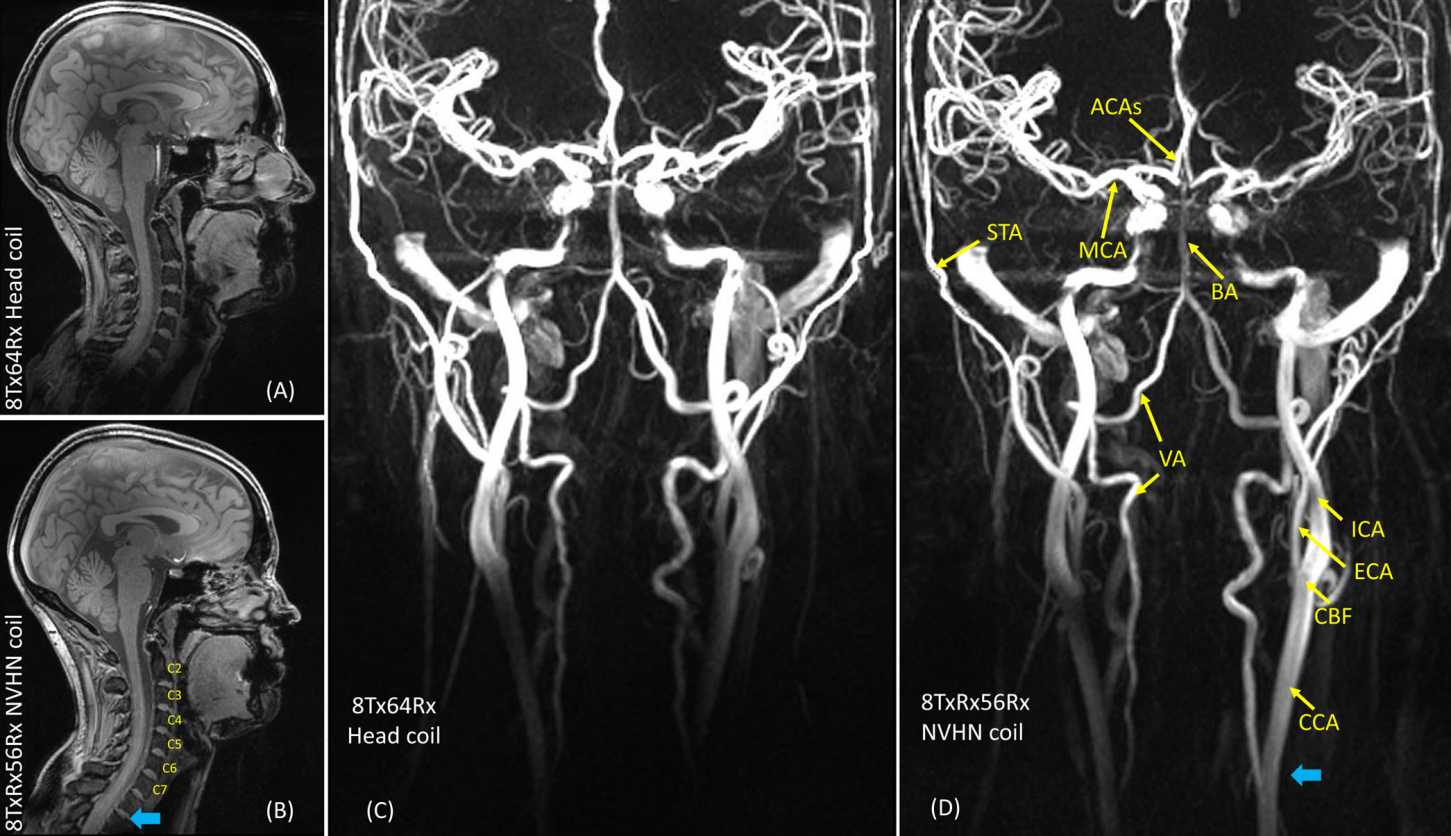
In vivo images of healthy volunteer acquired with NVHN and reference head coil. (A,B) FLASH sequence in sagittal midplane. (C,D) PC‐MRA in coronal midplane. ACA, anterior cerebral artery; BA, basilar artery; CBF, carotid bifurcation; CCA, common carotid artery; ECA, external carotid artery; ICA, internal carotid artery; MCA, middle cerebral artery; STA, superficial temporal artery; VA, vertebral artery.

The in vivo $B_{1}^{+}$ map, T_2_‐weighted turbo spin echo, and MP2RAGE images in Figure 9 show that the NVHN coil has a better coverage in the neck compared with the head coil without compromising the coverage in the brain. High‐resolution T_1_‐weighted FLASH images (Figure 10A,B) further confirm the large field of view offered by the NVHN coil, covering the whole brain and the cervical spine. Anatomic details in the neck, such as the spinal cord, cerebrospinal fluid, intervertebral discs, and vertebral bodies, are well visualized. The PC‐MRA data angiography data in Figure 10C,D demonstrates improved proximal cover of neck vessels, such as carotid and vertebral arteries in the NVHN coil. In addition, more signal at the level of the carotid bifurcation enables more accurate detection of pathology, such as arteriosclerotic plaque and resulting stenosis. Visualization of intracranial vessels at the level of the Circle of Willis is equivalent to the head coil.

## 4 DISCUSSION

Numerous brain pathologies involve the posterior fossa and extend to the spinal cord, including multiple sclerosis. Assessment of the neck and intracranial vessels is important, especially for stroke imaging, where visualization of the atherosclerotic plaque at carotid bifurcations is fundamental. A 7T coil that covers both the brain and neck is required for this purpose. Vascular techniques like arterial spin labeling could find many applications in tumor, vascular, epilepsy, and dementia imaging at 7 T, but rely on $B_{1}^{+}$ excitation of the upper neck to label blood. However, to enable concurrent brain–cord and brain–neck MRI, the imaging field of view that can be achieved must be much larger than for brain‐only, cord‐only, or neck‐only MRI scans. HN coils presented in the literature for concurrent brain and cord imaging at 7 T require either 16 transmit channels or a combination of power splitters and phase shift networks.^29^, ^30^, ^31^ Furthermore, 16‐channel systems are not industry‐standard, and they are substantially more expensive. The proposed NVHN coil achieves extended longitudinal coverage of the brain, neck vessel, and cervical spine with a total of eight transmit channels using a novel 6 + 2 configuration.

We introduced several features to enhance patient comfort, although it imposed technical challenges. This includes two wide cutouts in the RF shield in front of the eye, splitting the receive array into two halves, and mounting the transmit array on sliding rails to ease patient positioning. Initial simulation results indicate that the 6‐channel array produces a similar $B_{1}^{+}$ efficiency in the brain as the 8‐channel array (Figure 1C). This gives us the freedom to move the remaining two channels to the neck. The phase excitation of the lower two channels was optimized to improve field coverage in the neck without compromising the field distribution in the head. The average $B_{1}^{+}$ distribution in the sagittal midplane of the HS phantom (Figure 6), while exciting the upper six channels and all eight channels, are 43.45 and 45.80 nT/V, respectively. This indicates that the available $B_{1}^{+}$ is uniformly distributed in the brain or the brain and cervical spine, depending on the chosen excitation. Hence, the coil can be operated in two modes: (i) exciting the upper 6 channels in the head coil mode and (ii) 8‐channel excitation corresponding to the NVHN coil mode. The comparison of the 8‐channel and 6‐channel array simulations (Figure 1C) indicated similar field strength in the head region. However, the measured transmit efficiency was 5.8% lower for the 6‐channel NVHN coil (Figure 6A,C). This can be attributed to the large decoupling inductors in the NVHN coil, which are modeled as lossless inductors.

In terms of SNR, a factor of 1.16 gain in central SNR was observed compared with an array in conventional transmit‐only receive‐only configuration. We attribute this to the lack of receive elements in the anterior half of the receive array, and the additional contribution comes from receiving with the transmit elements, which extends further forward in the feet direction. We have demonstrated this on three different head coil setups at 7 T with reported gains of up to 20%.^40^, ^41^ Despite having only six transmit elements in the upper row, the NVHN coil yields a signal homogeneity in the brain region (Figures 9 and 10) that is comparable to the reference head coil with eight transmit elements, where a fixed‐shim $B_{1}^{+}$ is used in each case. PC‐MRA images of the volunteer in Figure 10C,D demonstrate good visualization of the blood vessels in the neck, including internal, external, and common carotid arteries. The in vivo images demonstrated that the proposed NVHN coil delivers images of the diagnostic quality of the brain and upper cord in a single acquisition. Our 8TxRx56Rx dual‐function NVHN coil demonstrates extended coverage in both phantom and in vivo imaging with higher (i) $B_{1}^{+}$ efficiency and (ii) SNR in the neck without compromising the brain, compared with the conventional 8‐channel head coil. The 7T NVHN coil, designed for concurrent brain, upper spine and vascular imaging, will extend the high‐resolution capability of 7T MRI to the routine diagnosis of pathologies affecting the cord, soft tissues, and vessels in the HN, in addition to the brain supratentorial and infratentorial, in clinical practice.

The next step involves improving both the mechanical and electrical design of the coil. The mechanical design of such a coil presents challenges owing to the substantial anatomical variations. Unlike a conventional head coil, an NVHN coil housing must be designed to allow individuals with both long and short necks to be optimally positioned inside the coil. At 7 T, this task is further complicated by the presence of the surrounding transmit array. In our current implementation, the NVHN coil is unable to provide comprehensive coverage in the neck region for individuals whose anatomical structure does not align with the intended positioning, particularly those with shorter necks unable to reach the apex of the receiver helmet (Figure S1). Therefore, further refinement of the mechanical design of the housing is imperative to comfortably accommodate a broader spectrum of the population. In terms of electrical design, routing RF cables and direct‐current wires through the transmit field to feed the receive elements in the neck region is quite challenging due to the extended posterior receive segment. We anticipate that further refinement on the implementation of the cables and wires within the transmit field with the addition of RF traps on the system cables will make the receive array more transparent to the transmit array. Adoption of the overlapped design in the transmit array, instead of the gapped design, appeared to enhance the extent of field coverage in the lower brain region (Figure S2). Further refinement of the dimensions of the lower two elements is essential to enhance the field produced in the cervical spine (Figure 9B). To the best of our knowledge, the proposed design is the first of its kind dual‐row design, in which we aim to achieve equivalent transmit performance of an 8‐channel array with six channels in the upper row, allowing the use of two additional channels to extend the longitudinal coverage. All results presented here were acquired in the pseudo‐CP configuration. Future work will exploit the pTx capability of the coil and further refine the design of the lower row to improve the transmit excitation.

## 5 CONCLUSION

This study presented the optimization, construction, and characterization of a novel NVHN coil with 8TxRx integrated with a 56Rx array. This new NVHN coil significantly improves the $B_{1}^{+}$ field distribution (factor of 2.9) and SNR (factor of 2.18) in the cervical spine region. The transceiver functionality of the array increases the central SNR by a factor of 1.16 and demonstrates a similar g‐factor in the brain region for different 1D and 2D acceleration factors. In vivo images confirm the coil's capability to produce diagnostic‐quality combined brain and cervical spine images in a single acquisition. Future work will focus on improving the electrical and mechanical design of the coil and investigate its RF shimming and pTx capabilities.

## CONFLICT OF INTEREST

Shajan Gunamony is a shareholder in MR CoilTech Limited, which is involved in the development of RF coils. However, this work was carried out at the University of Glasgow, and the research was funded by UKRI and other grants awarded to the University of Glasgow.

## Supporting information


**Figure S1.** Positioning of shorter neck subject in NVHN; (A) and Siemens 3T head/neck coil (B). (C) MP2RAGE of the subject obtained with NVHN coil at the sagittal midplane.
**Figure S2.** Combined $B_{1}^{+}$ map in the sagittal midplane of the HS phantom in CP mode of eight‐channel transmit array with overlapped loops and six‐channel transmit array (B) with gapped design with (B) and overlapped design (C).
