# Supplementary material for: Simultaneous whole‐brain and cervical spine imaging at 7 T using a neurovascular head and neck coil with 8‐channel transceiver array and 56‐channel receiver array
Source: Magn Reson Med. 2025 Jan 29;94(1):386–400. doi: 10.1002/mrm.30450 (PMC12021322; doi:10.1002/mrm.30450)
Supplement: Supplementary file 1 — Figure S1. Positioning of shorter neck subject in NVHN; (A) and Siemens 3T head/neck coil (B). (C) MP2RAGE of the subject obtained with NVHN coil at the sagittal midplane. Figure S2. Combined B1+ map in the sagittal midplane of the HS phantom in CP mode of eight‐channel transmit array with overlapped loops and six‐channel transmit array (B) with gapped design with (B) and overlapped design (C). [file MRM-94-386-s001.docx]

Supplementary Figures


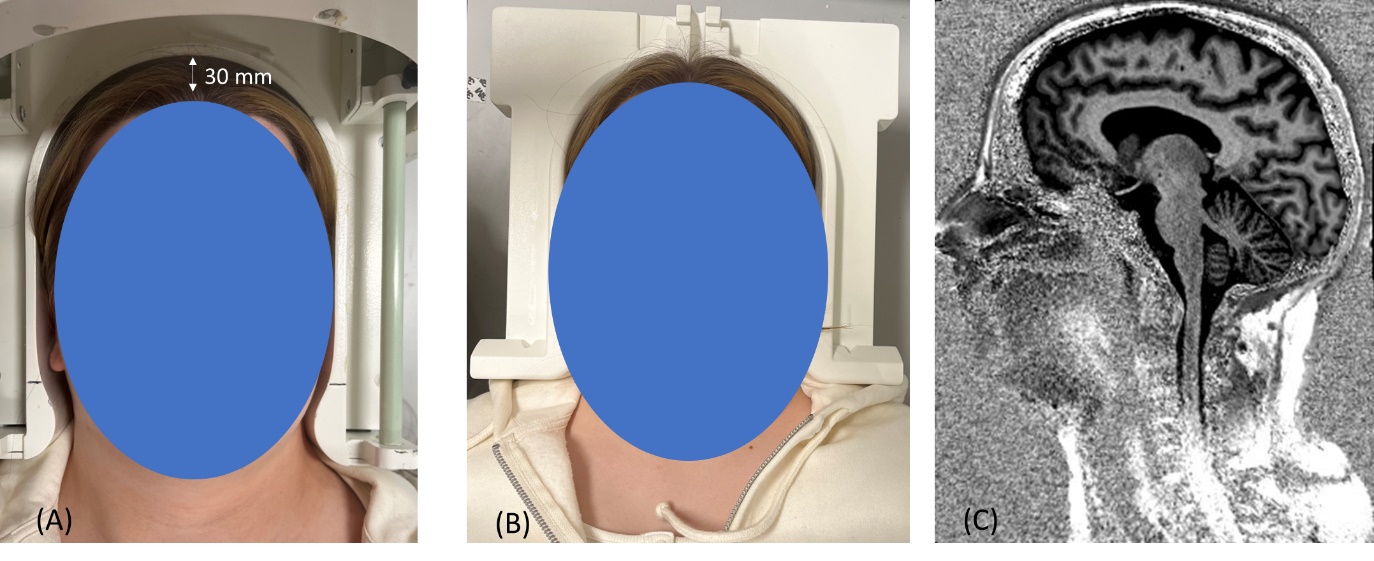


Figure S1. Positioning of shorter neck subject in (A) NVHN and (B) Siemens 3T head/neck coil. (C) MP2RAGE of the subject obtained with NVHN coil at the sagittal midplane.


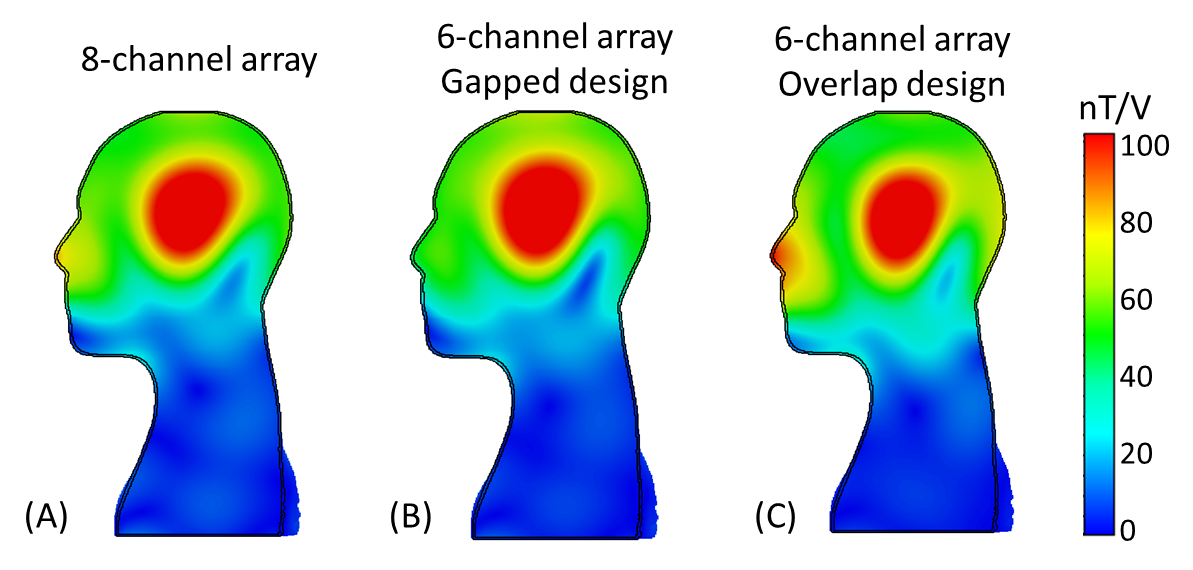


Figure S2. Combined $B_{1}^{+}$ map in the sagittal midplane of the HS phantom in CP mode of (A) 8-channel transmit array with overlapped loops and 6-channel transmit array with (B) gapped design, and (C) overlapped design.
